# The different role of YKL-40 in glioblastoma is a function of MGMT promoter methylation status

**DOI:** 10.1038/s41419-020-02909-9

**Published:** 2020-08-21

**Authors:** Wei-jun Chen, Xiang Zhang, Hua Han, Jian-nan Lv, En-ming Kang, Yu-lian Zhang, Wei-ping Liu, Xiao-sheng He, James Wang, Gui-huai Wang, Yan-bing Yu, Wei Zhang

**Affiliations:** 1 Department of Neurosurgery, Xijing Hospital, The Fourth Military Medical University, Xi’an, Shaanxi PR China; 2 Department of Emergency Medicine, Jinling Hospital, Medical School of Nanjing University, Nanjing, PR China; 3 Department of Medical Genetics and Developmental Biology, The Fourth Military Medical University, Xi’an, Shaanxi PR China; 4 Department of Neurosurgery, China-Japan Friendship Hospital, Beijing, PR China; 5 Department of Neurosurgery, Tsinghua Changgung Hospital, School of Clinical Medicine, Tsinghua University, Beijing, PR China

**Keywords:** Cancer stem cells, CNS cancer

## Abstract

Inter- and intratumoral heterogeneity is a hallmark of glioblastoma (GBM) that facilitates recurrence, treatment resistance, and worse prognosis. O^6^-methylguanine-DNA methyltransferase (*MGMT*) promoter methylation is a significant prognostic marker for Temozolomide (TMZ) resistance in GBM patients. *YKL-40* is a molecular marker for the mesenchymal subtype of GBMs and is responsible for TMZ resistance. However, underlying mechanisms by which MGMT epigenetics impacts patient outcomes and the function of YKL-40 are not fully determined. Herein, we performed in vitro and in vivo experiments, six human *IDH1/2* wild-type glioblastoma stem-like cells (GSCs) were established and studied to further determine a potential interaction of YKL-40 and MGMT promoter methylation. We demonstrated that *YKL-40* functioned differently in human *IDH1/2* wild-type GSCs. In *MGMT* promoter-methylated (*MGMT-m*) GSCs, it acted as a tumor suppressor gene. On the other hand, in *MGMT* promoter-unmethylated (*MGMT-um*) GSCs, it promoted tumorigenesis. Notably, the reason that *YKL-40* played different roles in GSCs could not be interpreted by the molecular classification of each GSCs, but is a function of *MGMT* promoter methylation status and involves the *RAS–MEK–ERK* pathway. *YKL-40* mediated TMZ sensitivity by activating DNA damage responses (DDRs) in *MGMT-m* GSCs, and it mediated resistance to TMZ by inhibiting DDRs in *MGMT-um* GSCs. Our report demonstrated that *MGMT* promoter methylation status might influence a gene’s function in human cancer. Moreover, our data also highlight the point that gene function should be investigated not only according to the molecular tumor classification, but also the epigenetic signature.

## Introduction

Glioblastoma (GBM) is the most lethal and common primary malignant brain tumor in adults. Standard therapy for GBM consists of surgery followed by radiotherapy and chemotherapy with the alkylating agent temozolomide (TMZ). However, the overall survival is <2 years^1^.

*YKL-40*, also known as *CHI3L1*, is a 40-kDa secreted glycoprotein that is expressed by both tumor cells and their surrounding tumor-infiltrating macrophages^2^. In patients with solid tumors, elevated serum/plasma YKL-40 was significantly associated with poor overall survival. These tumors included: lung cancer, breast cancer, gastrointestinal cancer, ovarian cancer, urologic cancer, prostate cancer, malignant melanoma, and squamous cell carcinoma of the head and neck^2–5^. Moreover, elevated serum/plasma concentration of YKL-40 was an independent prognostic variable indicating a short recurrence-free interval and short overall survival^2^. In GBM, based on the molecular classification of GBMs in The Cancer Genome Atlas, *YKL-40* is recognized as a marker of the mesenchymal subtype^6,7^. It was differentially expressed in TMZ-resistant GBM cells and was responsible for TMZ resistance^8^. A limited number of studies have shown that YKL-40 plays a vital role in glioma cell proliferation through activation of the MAPK and AKT pathway^2^. However, a more detailed understanding of the role of YKL-40 in GBM remains unclear.

O^6^-methylguanine-DNA methyltransferase (*MGMT*) promoter methylation predicts a favorable response to alkylating chemotherapy, such as TMZ, in GBM patients^9–16^. In addition, *MGMT* promoter methylation status not only mitigates sensitivity or resistance to TMZ, but may also shift the GBM mutational spectrum in the context of alkylating treatment, which may facilitate the emergence of secondary resistance to alkylating agents^17–19^. All of the mechanisms by which *MGMT* promoter methylation impacts the survival of GBM patients are not fully understood.

To understand the function of *YKL-40* in the maintenance of stemness and tumorigenicity of GBM stem-like cells (GSCs), we performed in vitro and in vivo experiments. We demonstrated that *YKL-40* impact on GSCs is a function of *MGMT* promoter methylation status and involves the *RAS–MEK–ERK* pathway. *YKL-40* mediated TMZ sensitivity by activating DNA damage responses (DDRs) in *MGMT* promoter-methylated (*MGMT-m*) GSCs, and it mediated resistance to TMZ by inhibiting DDRs in *MGMT* promoter-unmethylated (*MGMT-um*) GSCs. Our report demonstrated that *MGMT* promoter methylation status might influence a gene’s function in human cancer.

## Results

### Isolation and characterization of six GSC cultures from human GBM specimens

**Fig. 1:**
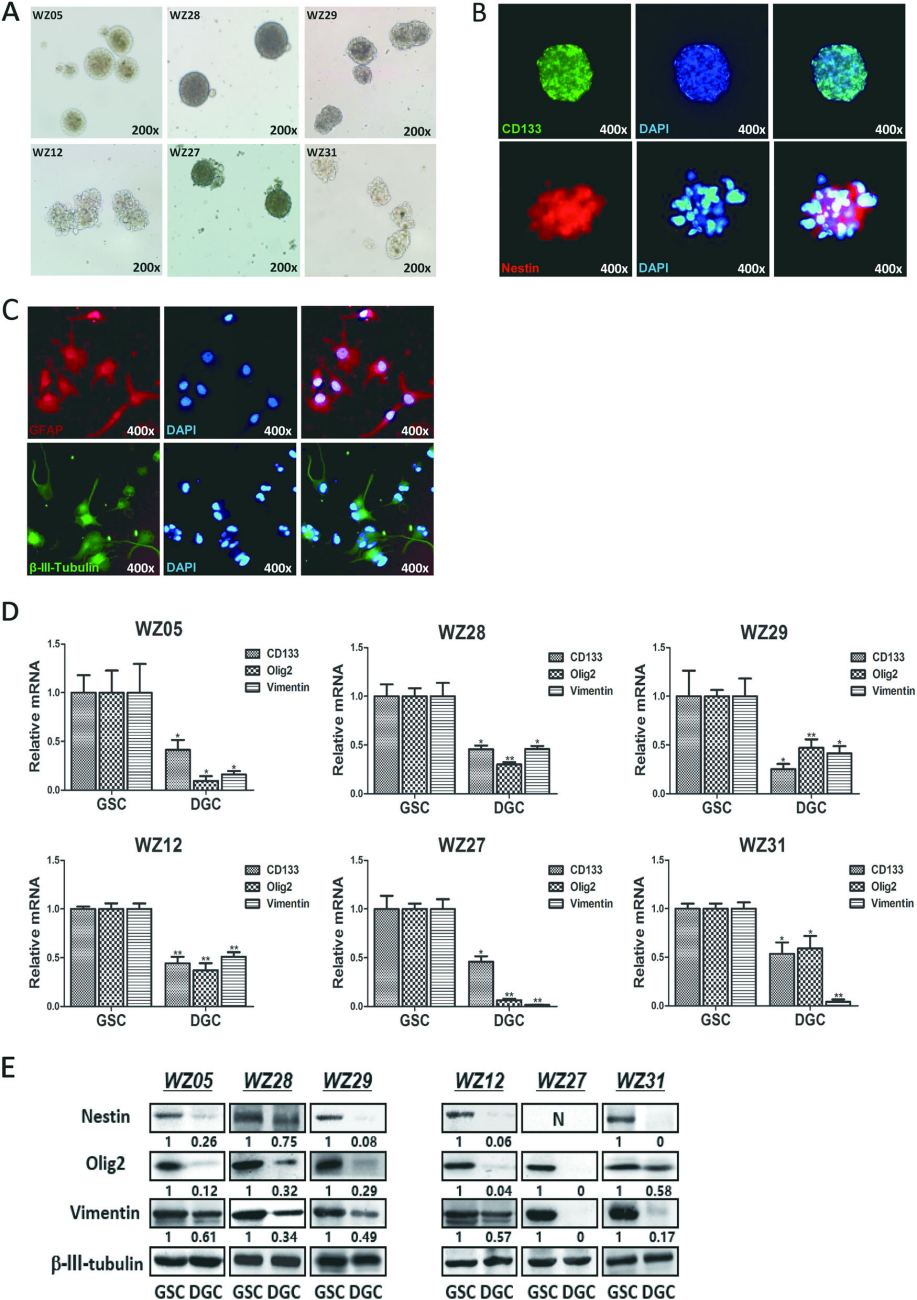
Characterization of six GBM-derived cancer stem cells. **a** Typical appearance of a “neurosphere” structure of corresponding GSCs grown in serum-free culture conditions (WZ05, WZ28, and WZ29, upper; WZ12, WZ27, and WZ31, down). Magnification, ×200. **b** Representative images of the neural stem/progenitor marker *CD133* (green) and *Nestin* (red) staining. Magnification, ×400. **c** Representative images of *GFAP* (red) and *Tubulin* (green) staining after the induction of differentiation. Magnification, ×400. **d** Quantitative PCR to measure the transcript levels of *CD133*, *Olig2*, and *Vimentin* in DGCs compared with GSCs (*P < 0.05; **P < 0.01). **e** Western blotting analyses to measure the protein levels of *Nestin*, *Olig2*, and *Vimentin* in DGCs compared with GSCs (*P < 0.05; **P < 0.01). *Nestin* was not expressed in WZ27. The ratio has been normalized by *β-III-tubulin*.

To identify the role of *YKL-40* in GSCs, we first established GSC cultures from GBM specimens as previously described^20^. Characteristics of patients were described (Table 1). Tumor tissue cultures yielded typical neurosphere-like structures within 5 days (Fig. 1a). All tumors were *IDH1/2* wild-type primary GBMs, were stable, and could be passaged for more than 3 months. Immunofluorescence staining revealed that the majority of the cells from these cultures were positive for *CD133* and *Nestin*, which are markers for neural stem/progenitor cells (Fig. 1b). To examine the capability of the cells to differentiate into neural cell lineages, we induced the cells to differentiate by exposing them to serum. Differentiated GSCs (DGCs) were grown as adherent monolayers and were positive for astroglial (*GFAP*) and neuronal (*βIII-tubulin*) markers (Fig. 1c). Compared with their GSC counterparts, the transcription levels of *CD133*, *Olig2*, *Vimentin*, and protein expression levels of *Nestin*, *Olig2*, *Vimentin* were significantly decreased in all six DGCs (Fig. 1d, e).

**Table 1:** Characteristics of patients with primary GBM.

Patient number	Age (Year)	Sex	Location	Histological Diagnosis	Overall survival (month)
WZ05	39	M	Frontal	GBM	13
WZ12	40	M	Tempoal	GBM	11
WZ27	30	F	Frontal	GBM	9
WZ28	49	M	Temporal	GBM	14
WZ29	51	F	Frontal	GBM	12
WZ31	58	F	Frontal	GBM	6

### *YKL-40* played different role in the biological properties of GSCs

**Fig. 2:**
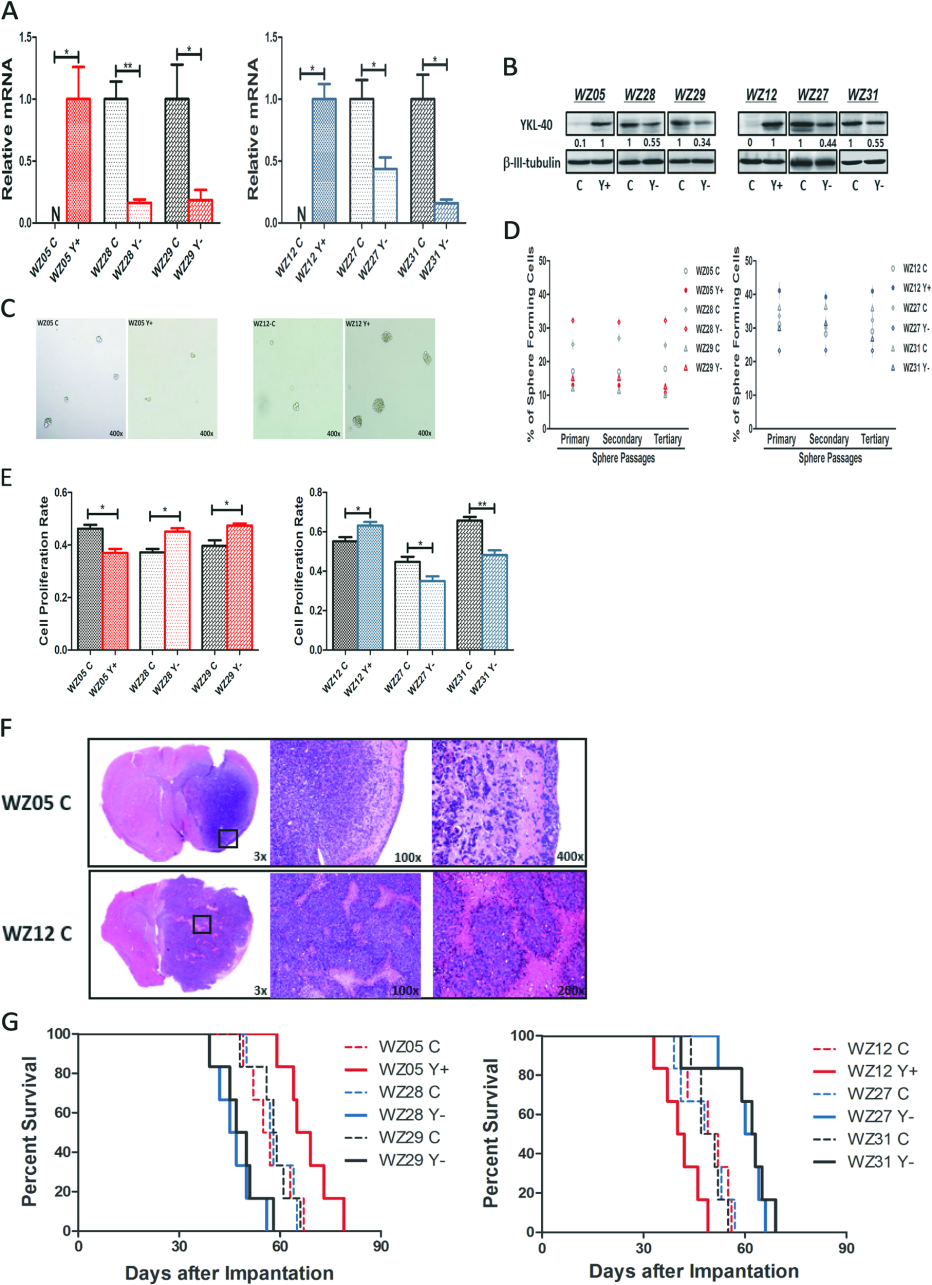
Self-renewal and proliferation properties in GSC Y+ or Y−. **a** Quantitative PCR to validate the transcript levels of *YKL-40* overexpression in WZ05 and WZ12 GSCs (Y+) and *YKL-40* gene silencing in WZ28, WZ29, WZ27, and WZ31 GSCs (Y−) (*P < 0.05; **P < 0.01). **b** Western blotting analyses to validate the protein levels of *YKL-40* overexpression in WZ05 and WZ12 GSCs (Y+) and *YKL-40* gene silencing in WZ28, WZ29, WZ27, and WZ31 GSCs (Y−) (*P < 0.05; **P < 0.01). The ratio has been normalized by *β-III-tubulin*. **c** Contrast field image of representative GSC spheres in self-renewal. Left, WZ05 and WZ05 Y+ GSCs; Right, WZ12 and WZ12 Y+ GSCs. Magnification, ×400. **d** Data points demonstrated the percentage of single cells capable of serial sphere formation in three consecutive passages in serum-free conditions. Self-renewal properties of GSC Y+ or Y− were comparable to corresponding control of GSC. **e** Bar graph indicating the proliferation rates measured by BrdU incorporation. Proliferation of GSC Y+ or Y− was compared with the controls of each GSC (*P < 0.05; **P < 0.01). **f** Representative magnified coronal sections of xenografts stained with H&E demonstrate the characteristic features of GBM. **g** Athymic mice intracerebrally implanted with 1 × 10^5^ GSCs. Mice were then monitored for survival. *n* = 6 mice per group. The survival curve depicted the in vivo tumor-propagating potential of GSCs Y+ or Y− compared with controls of each GSC (WZ05 vs. WZ05 Y+ *P* = 0.02; WZ28 vs. WZ28 Y− *P* = 0.005; WZ29 vs. WZ29 Y− *P* = 0.02; WZ12 vs. WZ12 Y+ *P* = 0.03; WZ27 vs. WZ27 Y− *P* = 0.006; WZ31 vs. WZ31 Y− *P* = 0.01).

To explore the function of *YKL-40* in GSCs, we measured baseline levels of *YKL-40* in our established GSCs. We observed that WZ05 and WZ12 did not express and secrete *YKL-40* at a detectable level, and the remaining GSCs, i.e., WZ27, WZ28, WZ29, and WZ31, expressed and secreted high levels of YKL-40 (Fig. S1). To investigate the biological function of *YKL-40* in GSCs, we modified by overexpressing it in WZ05, WZ12 GSCs (Y+) and knocking it down in WZ27, WZ28, WZ29, and WZ31 GSCs (Y−). The effect of overexpression or gene silencing was confirmed (Fig. 2a, b). To explore self-renewal and cell proliferation capabilities in vitro, single-cell sphere-formation and the BrdU assay were performed in both control and modified cells, respectively. *YKL-40* significantly enhanced the self-renewal and cell proliferation capabilities of WZ12, WZ27, and WZ31 (Fig. 2c–e, right). However, we observed that *YKL-40* impaired the self-renewal and cell proliferation abilities in WZ05, WZ28, and WZ29 GSCs (Fig. 2c–e, left). We further investigated these findings in an orthotopic xenograft tumor model in vivo (Fig. 2f, g). Interestingly, the longer overall survival of WZ05, WZ28, and WZ29 was observed both in patients and in vivo when compared with the overall survival of WZ12, WZ27 and WZ31, even though without statistically significance. This indicated that our GSCs could recapitulate the tumorigensis of their parental tumors. Furthermore, we observed that *YKL-40* overexpression significantly prolonged the overall survival of nude mice implanted with WZ05 Y+ GSCs while knockdown of *YKL-40* shortened the overall survival of nude mice implanted with WZ28 Y− and WZ29 Y− GSCs (Fig. 2g, left), suggesting a inhibitory role of *YKL-40* in tumor initiation or progression in these GSCs (WZ05 vs. WZ05 Y+ *P* = 0.019; WZ28 vs. WZ28 Y− *P* = 0.004; WZ29 vs. WZ29 Y− *P* = 0.02). In contrast, *YKL-40* overexpression significantly shortened the overall survival of nude mice implanted with WZ12 Y+ GSCs, knockdown of *YKL-40* prolonged the overall survival of nude mice implanted with WZ27 Y− and WZ31 Y− GSCs (Fig. 2g, right), suggesting a positive role of *YKL-40* in tumor initiation or progression in these GSCs (WZ12 vs. WZ05 Y+ *P* = 0.032; WZ27 vs. WZ27 Y− *P* = 0.005; WZ31 vs. WZ31 Y− *P* = 0.011).

**Fig. 3:**
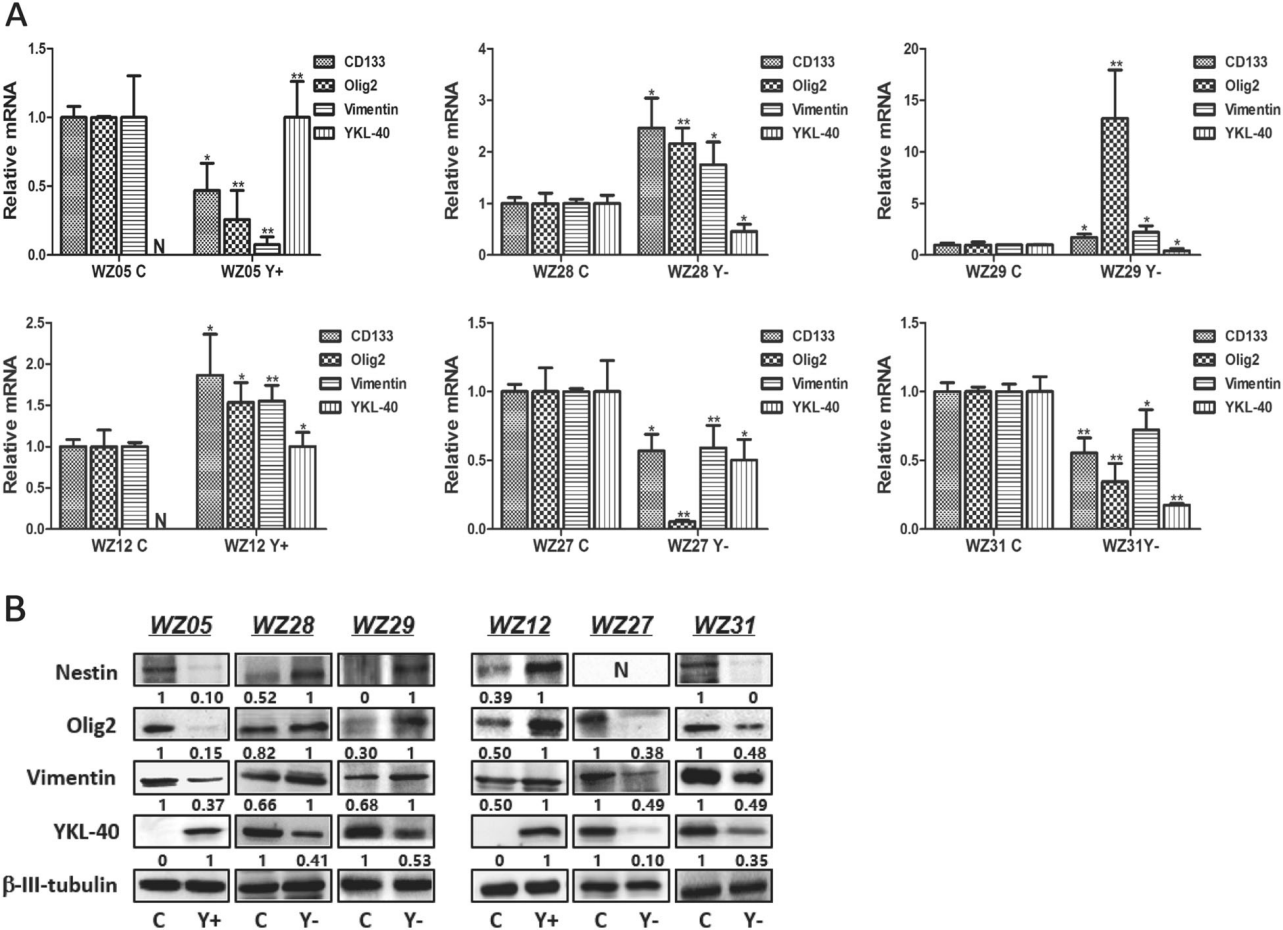
Molecular changes of stemness in GSC Y+ or Y−. **a** Quantitative PCR to measure the transcript levels of *CD133*, *Olig2*, *Vimentin*, and *YKL-40* in GSC Y+ or Y− compared with controls of each GSC (*P < 0.05; **P < 0.01). **b** Western blot analyses measuring the protein expression levels of *Nestin*, *Olig2*, *Vimentin*, and *YKL-40* in GSC Y+ or Y− compared with the controls for each GSC. The ratio has been normalized by *β-III-tubulin*.

Next we investigated changes in the stemness of each GSC caused by *YKL-40* overexpression or silencing. The transcript levels of *CD133*, *Olig2*, and *Vimentin* were decreased in WZ05 Y+, WZ27 Y−, and WZ31 Y− GSCs (*P* < 0.05) but increased in WZ12 Y+, WZ28 Y− and WZ29 Y− GSCs (*P* < 0.05) (Fig. 3a). The protein expression level of *Nestin*, *Olig2*, and *Vimentin* was decreased in WZ05 Y+, WZ27 Y−, and WZ31 Y− GSCs (*P* < 0.05) but increased in WZ12 Y+, WZ28 Y−, and WZ29 Y− GSCs (*P* < 0.05) (Fig. 3b).

### *YKL-40* played different role in GSCs involved the *RAS–MEK–ERK* pathway

**Fig. 4:**
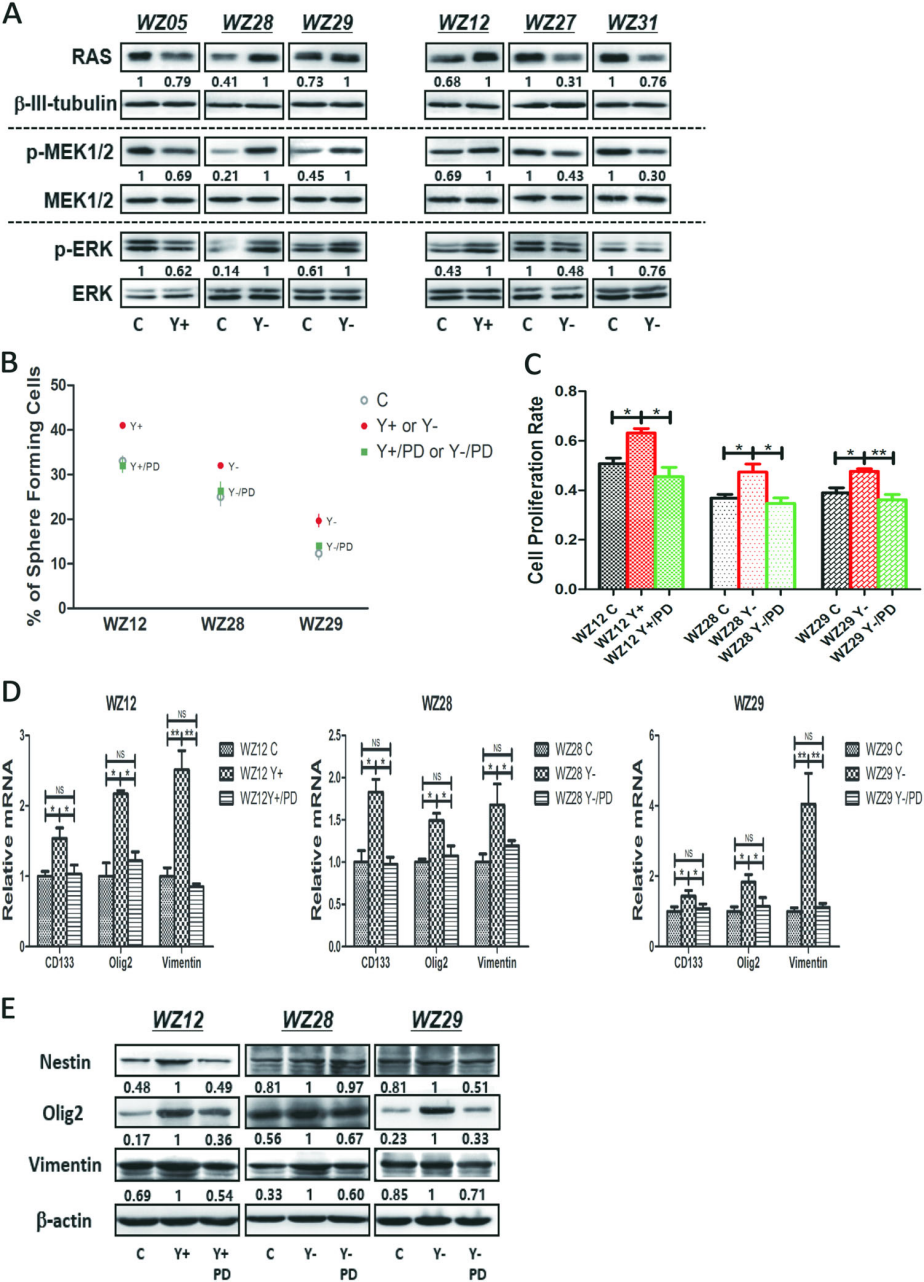
The different role of *YKL-40* in GSCs involved the *RAS–MEK–ERK* pathway. **a** Western blot analyses to measure the protein expression levels of *RAS*, *β-tubulin*, *pMEK1/2*, *MEK1/2*, *pERK1/2,* and *ERK1/2* in GSC Y+ or Y− compared with controls of each GSC. The ratio has been normalized by *β-III-tubulin*. **b** Following the addition of the *ERK1/*2-specific inhibitor PD98059, data points demonstrated the percentage of single cells capable of sphere formation in serum-free conditions in GSC Y+ or Y− compared with controls of each GSC. PD = PD98059. **c** With the addition of the *ERK1/2*-specific inhibitor PD98059, the bar graph indicated the proliferation rates measured using BrdU incorporation in GSC Y+ or Y− compared with controls of each GSC (*P < 0.05; **P < 0.01). PD demonstrated PD98059. No significant difference was observed between WZ12 C vs. WZ12 Y+/PD, WZ28 C vs. WZ28 Y-/PD, WZ29 C vs. WZ29 Y-/PD. **d** With the addition of the *ERK1/*2-specific inhibitor PD98059, the bar graph indicated the transcript levels of *CD133*, *Olig2*, *Vimentin* in GSC Y+ or Y− compared with controls of each GSC (*P < 0.05; **P < 0.01). **e** With the addition of the *ERK1/*2-specific inhibitor PD98059, western blotting analyses were performed to measure the protein expression levels of *Nestin*, *Olig2*, and *Vimentin* in GSC Y+ or Y− compared with controls of each GSC (*P < 0.05; **P < 0.01). The ratio has been normalized by *β-actin*.

Our previous study showed that the *MAPK* pathway was altered by *YKL-40* silencing in U87 cells and primary astrocytoma cells. We hypothesized that the *MAPK* pathway could be involved in the different role of *YKL-40* in GSCs. We observed that overexpression of *YKL-40* decreased the expression of *RAS*, *pMEK1/2*, and *pERK1/2* in WZ05. Notably, knockdown of *YKL-40* increased the expression of *RAS*, *pMEK1/2,* and *pERK1/2* in WZ28 and WZ29 (Fig. 4a, left). Conversely, overexpression of *YKL-40* increased the expression of *RAS*, *pMEK1/2,* and *pERK1/2* in WZ12, and its knockdown decreased the expression of *RAS*, *pMEK1/2,* and *pERK1/2* in WZ27 and WZ31 (Fig. 4a, right). Furthermore, by using single-cell sphere formation and BrdU assays, we demonstrated that PD98059, a specific inhibitor of *ERK1/2*, significantly inhibited the self-renewal and cell proliferation capabilities of GSCs in WZ12 Y+/PD, WZ28 Y−/PD, and WZ29 Y−/PD GSCs (Fig. 4b, c). Accordingly, results of quantitative PCR (Fig. 4d) and western blotting analyses (Fig. 4e) showed that the transcript levels of *CD133*, *Olig2*, *Vimentin* and protein expression level of *Nestin*, *Olig2*, *Vimentin* were significantly rescued in WZ12 Y+/PD, WZ28 Y−/PD, and WZ29 Y−/PD GSCs compared with their counterparts.

### *MGMT* promoter methylation status, but not GBM molecular subtypes, is associated with the different role of *YKL-40* in GSCs

**Fig. 5:**
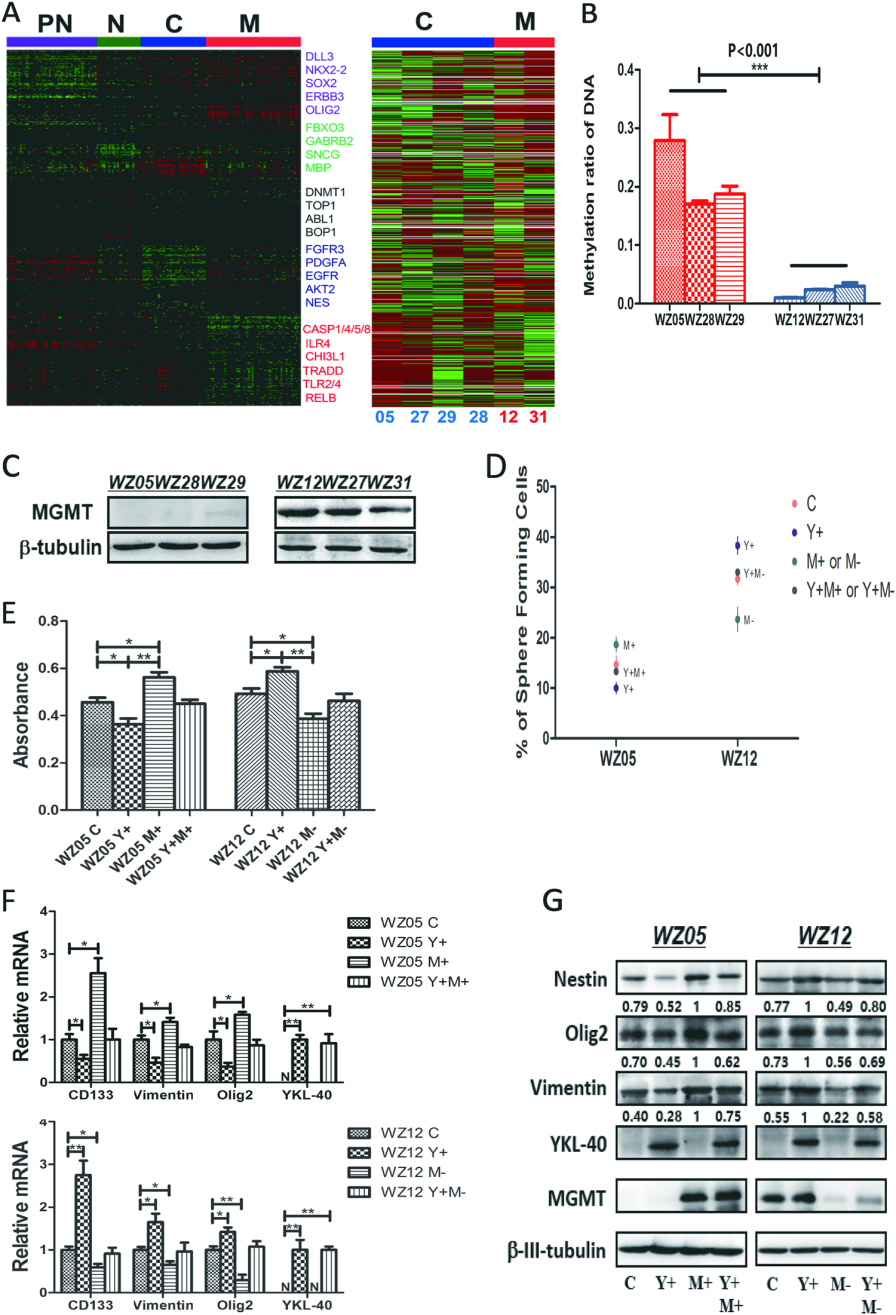
*MGMT* promoter methylation status associated with the different role of *YKL-40* in GSCs. **a** RNA-seq data demonstrating the molecular subtypes of six GSCs. Left, the core set of 173 TCGA GBM samples were ordered based on subtype predictions using the predictive 840-gene list, according to Verhaak^6^; right, ordered gene expression for six GSCs. Samples were ordered based on their predicted identity using the 840-gene list. **b** MSP indicated the *MGMT* promoter methylation status of GSCs. Each GSC was measured in triplicate. **c** Western blot analyses were performed to quantify the protein expression levels of *MGMT* in GSCs. **d** Following overexpression of *YKL-40* (Y+) or/and *MGMT* (M+) in WZ05 GSC or overexpression of *YKL-40* (Y+) or/and knockdown *MGMT* (M−) in WZ12 GSC, the data points demonstrate the percentage of single cells capable of sphere formation in serum-free conditions in each GSC. (WZ05 C vs. WZ05 Y+ *P* = 0.037; WZ05 C vs. WZ05 M+ *P* = 0.022; WZ05 Y+ vs. WZ05 M+ *P* = 0.003; WZ05 Y+ vs. WZ05 Y+M+ *P* = 0.039; WZ05 M+ vs. WZ05 Y+M+ *P* = 0.015; WZ12C vs. WZ12 Y+ *P* = 0.029; WZ12C vs. WZ12 M− *P* = 0.027; WZ12 Y+ vs. WZ12 M− *P* = 0.001; WZ12 Y+ vs. WZ12 Y+M− *P* = 0.022; WZ12 M− vs. WZ12 Y+M− *P* = 0.016; no significant difference was observed between WZ05 C vs. WZ05 Y+M+, WZ12C vs. WZ12 Y+M−). **e** Following overexpression of *YKL-40* (Y+) or/and *MGMT* (M+) in WZ05 GSC or overexpression of *YKL-40* (Y+) and/or knockdown *MGMT* (M−) in WZ12 GSC, the bar graph indicated the proliferation rates measured using BrdU incorporation in each GSCs (*P < 0.05; **P < 0.01). No significant difference was observed between WZ05 C vs. WZ05 Y+M+, WZ12 C vs. WZ12 Y+M−. **f** Quantitative PCR was used to quantify the transcript levels of *CD133*, *Olig2*, *Vimentin*, and *YKL-40* in the WZ05 and WZ12 GSCs (*P < 0.05; **P < 0.01). No significant difference was observed between WZ05 C vs. WZ05 Y+M+, WZ12 C vs. WZ12 Y+M− for *CD133*, *Olig2,* and *Vimentin*. **g** Western blot analyses confirmed the protein expression level of *Nestin*, *Olig2*, *Vimentin*, *YKL-40*, and *MGMT* in the WZ05 and WZ12 GSCs. The ratio has been normalized by *β-III-tubulin*. No significant difference was observed between WZ05 C vs. WZ05 Y+M+, WZ12 C vs. WZ12 Y+M− for *Nestin*, *Olig2*, and *Vimentin*.

To explore what could distinguish the difference in the function of *YKL-40* in GSCs, we performed RNA-Seq to identify the molecular subclasses of each GSC as previously described^6^. These results indicated that WZ05, WZ27, WZ28, and WZ29 belonged to the classical subtype, whereas WZ12 and WZ31 were of the mesenchymal subtype (Fig. 5a). Thus, the molecular subtypes could not distinguish the different role of *YKL-40* in GSCs.

As an epigenetic event, *MGMT* promoter methylation is a favorable prognostic marker, and patients with tumors exhibiting *MGMT* promoter methylation have a survival benefit from receiving combined radiotherapy and temozolomide chemotherapy^9,10^. We therefore hypothesized that the *MGMT* promoter methylation status of GSCs might be associated with the different functions of *YKL-40*. To investigate this hypothesis, we performed methylation-specific PCR (MSP) to determine the *MGMT* promoter methylation status of each GSC. MSP is one of the most commonly used DNA-based methods for detecting promoter methylation because of its simplicity and cost-effectiveness^19^. We observed that the *MGMT* promoter was highly methylated in WZ05, WZ28, and WZ29 and was unmethylated in WZ12, WZ27, and WZ31 (Fig. 5b). Since *MGMT* promoter methylation patterns are associated with *MGMT* protein expression levels, we further examined *MGMT* protein expression in these GSCs using western blotting analyses. *MGMT* protein was highly expressed in WZ12, WZ27, and WZ31 but was undetectable in WZ05, WZ28, and WZ29 (Fig. 5c). MGMT overexpressing in WZ05 (WZ05 M+) or silencing in WZ12 (WZ12 M−) could enhance or inhibit self-renewal and cell proliferation ability in vitro when compared with their control group, respectively (Fig. 5d, e). We further explored whether the gain or loss of *MGMT* might reverse the effect caused by overexpressing of *YKL-40* in WZ05 and WZ12. Notably, by co-overexpressing *YKL-40* and *MGMT* in WZ05 GSC (WZ05 Y+M+), it significantly reversed the inhibited effect of WZ05 Y+ alone by enhancing self-renewal and cell proliferation in vitro (Fig. 5d, e). This finding was confirmed by examining the mRNA transcription and protein expression levels of stemness markers (Fig. 5f, g). In addition, by overexpressing *YKL-40* and knocking down the expression of *MGMT* in WZ12 GSC (WZ12 Y+M−), we observed that WZ12 Y+M− reversed the effect of WZ12 Y+ in vitro (Fig. 5d, e). The altered mRNA transcription and protein expression levels of stemness markers further confirmed the finding (Fig. 5f, g).

### *YKL-40* sensitized the response of TMZ in *MGMT-m* GSCs and accounted for TMZ resistance in *MGMT-um* GSCs

**Fig. 6:**
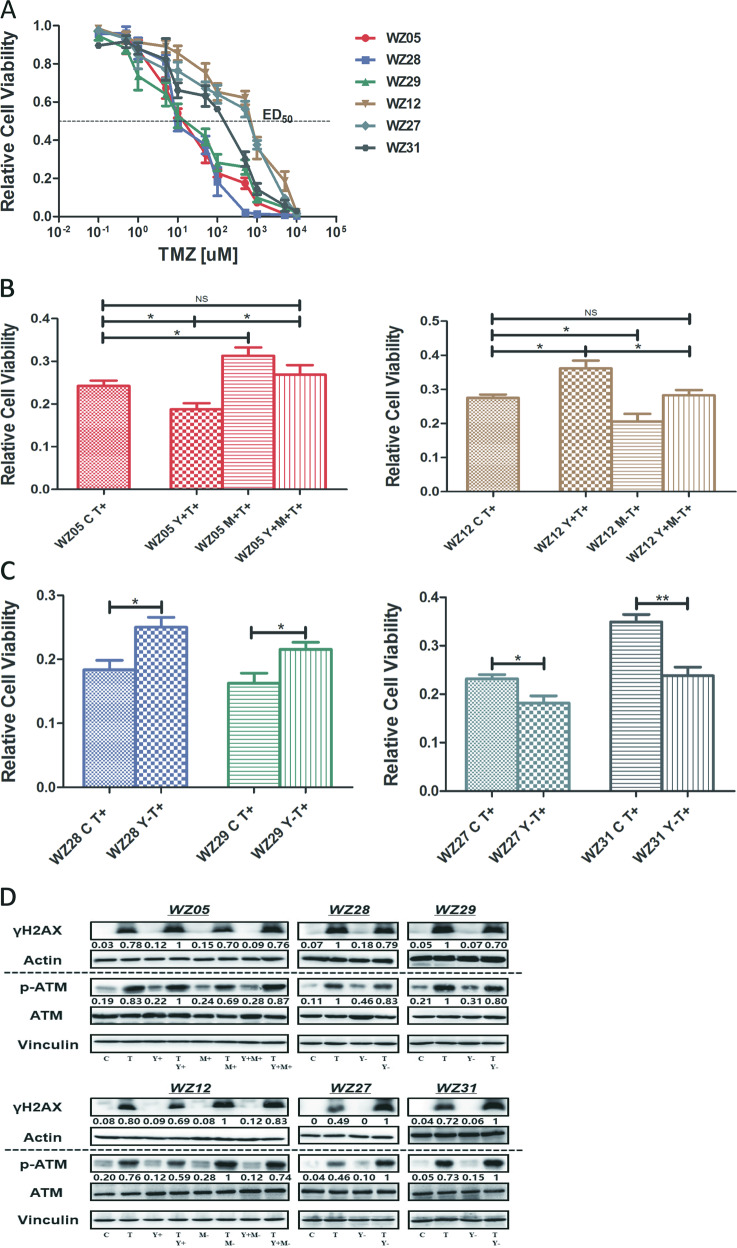
*YKL-40* sensitized the response of TMZ in *MGMT-m* GSCs and accounted for TMZ resistance in *MGMT-um* GSCs. **a** TMZ dose–response curves. GSCs were cultured in triplicate wells in vitro measured using BrdU 5 days after the addition of TMZ. Error bars represent 95% confidence intervals. Dotted line indicates 50% cell viability relative to mock-treated cells. TMZ temozolomide. **b** With the addition of ED50 TMZ to WZ05 and WZ12 series (T+), the bar graph indicated the proliferation rates measured by BrdU incorporation in each GSC (*P < 0.05; **P < 0.01). No significant difference was observed between WZ05 C T+ vs. WZ05 Y+M+T+, WZ12 C T+ vs. WZ12 Y+M−T+. **c** With the addition of ED50 TMZ to WZ28, WZ29, WZ27, and WZ31 GSCs (T+), the bar graph indicated the proliferation rates measured by BrdU incorporation in each GSCs (*P < 0.05; **P < 0.01). **d** Western blotting analyses were performed to quantify the protein expression levels of *γH2AX*, *Actin*, *p-ATM*, *ATM* in each GSC series. The ratio has been normalized by *Vinculin*.

To examine the effects of *YKL-40* on the sensitivity of GSCs to TMZ, we performed a cell viability assay as previously described^21^. The six GSCs exhibited variable sensitivities to TMZ (Fig. 6a), with three (WZ05, WZ28, and WZ29) being highly sensitive (ED50 < 30 µM) and three (WZ12, WZ27 and WZ31) being highly resistant (ED50 > 200 µM) (Table 2). As predicted, WZ05, WZ28, and WZ29, which are *MGMT-m*, were highly sensitive; WZ12, WZ27, and WZ31, which are *MGMT-um* cells, were highly resistant. Notably, in the *MGMT-m* group (WZ05, WZ28, and WZ29), *YKL-40* sensitized the response of GSCs to TMZ (Fig. 6b, c, left). By contrast, in the *MGMT-um* group (WZ12, WZ27, and WZ31), *YKL-40* contributed to GSCs resistance to TMZ (Fig. 6b, c, right). Furthermore, overexpression of *MGMT* could increase the resistance of WZ05 to TMZ, and co-overexpression of *MGMT* and *YKL-40* attenuated the resistance caused by overexpressing *MGMT* alone (Fig. 6b, left). Knocking down *MGMT* also decreased the resistance of WZ12 to TMZ, and overexpressing *YKL-40* and knocking down *MGMT* rescued the effect caused by *MGMT* silencing alone (Fig. 6b, right).

**Table 2:** Median-effect doses of TMZ in vitro.

GSC line	ED_50_: TMZ µM
WZ05	22
WZ28	10
WZ29	25
WZ12	900
WZ27	850
WZ31	250

It is well known that TMZ induces DDR, and phosphorylation of H2AX (*p-H2AX*) is closely associated with the process as a sensitive molecular marker of DNA double strand breaks (DSBs)^22^. Thus, we hypothesized that the effect of *YKL-40* on response to TMZ could be mediated via DDR. Western blotting results clearly demonstrated that *YKL-40* sensitized the response to TMZ in *MGMT-m* GSCs (WZ05, WZ28, WZ29) via an activation of *p-H2AX* and *p-ATM* (Fig. 6d). In *MGMT-um* GSCs (WZ12, WZ27, WZ31), *YKL-40* inhibited the activation of *p-H2AX* and *p-ATM* (Fig. 6d). Notably, overexpression of *MGMT* inhibited the activation of DDRs in TMZ-treated WZ05, and co-overexpression of *MGMT* and *YKL-40* attenuated the effect caused by overexpression of *MGMT* alone (Fig. 6d). Furthermore, knocking down of *MGMT* could induce DDRs in WZ12 to TMZ, and overexpressing YKL-40 and knocking down *MGMT* attenuated the effect caused by *MGMT* silencing alone (Fig. 6d).

Thus, these in vitro and in vivo experiments demonstrated that *MGMT* promoter methylation status of GSCs is associated with different roles of *YKL-40* in these cells. This supports the notion that a gene can act either as a tumor promoter or tumor suppressor gene depending on the epigenetic context.

## Discussion

We here demonstrated that the *YKL-40* gene, which is recognized as a molecular marker of the mesenchymal subtype of GBMs^6,7^, functioned differently in human *IDH1/2* wild-type GSCs. In *MGMT-m* GSCs, it acted as a tumor suppressor gene and sensitized GSCs’ response to TMZ by activating DDRs. On the other hand, in *MGMT-um* GSCs, it promoted tumorigenesis and accounted for TMZ resistance by inhibiting DDRs. Notably, the reason that YKL-40 played different roles in GSCs could not be interpreted by the molecular classification of each GSCs, but was closely related with their *MGMT* promoter methylation status. Our report demonstrated that *MGMT* promoter methylation status could influence a cancer-relevant gene’s function. These data also highlight the point that gene function should be investigated not only according to the molecular tumor classification, but also the epigenetic signature.

Based on prior work, we realized it is not by chance that *YKL-40*’s different role could not be interpreted by the molecular classification in GSCs. These molecular classifications of GBMs are based on the DNA and RNA profiles of bulk tumors mixed with various cell types, GSCs are only a subpopulation among those various cells^6,7,23,24^. Single-cell RNA-seq revealed that individual tumors contained a spectrum of GBM subtypes and hybrid cellular states^25^. Cells from the same tumor may harbor different mutations or epigenetic states. Therefore, molecular classification does not completely recapitulate intratumoral heterogeneity, which has been increasingly appreciated as a determinant of treatment resistance and tumor recurrence in GBM. Interestingly, a comprehensive approach involving DNA methylation-based classification of central nervous system tumors across all entities and age groups, indicated that a combined DNA methylation signature with histology and molecular tumor classification could improve the accuracy of diagnosis^26^. In another study it was demonstrated that epigenetic subtypes were significant independent predictors of survival in multivariate analysis in 1122 adult diffuse grade II, III, and IV gliomas^27^. All of these data emphasize the point that the epigenetic context is critical in neuropathology and in a patient’s prognosis. Specifically, our study demonstrates that the epigenetic context, in the form of MGMT promoter methylation status is critical for *YKL-40* gene function. Further experiments and more cases of GBM are required to validate our findings.

As a critical epigenetic event, promoter methylation of the DNA repair enzyme gene *MGMT* could compromise DNA repair mechanisms and increase sensitivity to TMZ. The value of *MGMT* promoter methylation for predicting a favorable response to alkylating chemotherapy in GBM has been established^9–16,19^. *MGMT* promoter methylation was identified as a positive prognostic marker for overall (21.2 vs. 14 months) and progression-free survival (8.7 vs. 5.7 months) in newly diagnosed GBM patients^12^. Our results provide new insights into the potential mechanisms by which GBM patients benefit from *MGMT* promoter methylation. In this study, we focused on the *YKL-40* gene only, more genes should be evaluated for their function in the context of *MGMT*-*m* vs. *MGMT-um*.

As a critical pathway in *IDH1/2* wild-type gliomas, alterations in the *RAS–MEK–ERK* signaling pathway have been detected in most cases^27^. This pathway plays a critical role in maintaining stemness and promoting tumorigenesis in GSCs^28^. It has been shown that *YKL-40* expression has a positive association with the expression of phosphor-*ERK1/2*, which is strongly correlated with a poor response to radiotherapy and poor clinical outcome^29^. Our previous study indicated that the vital role that *YKL-40* played in established glioma cell proliferation was through the activation of MAPK and AKT pathway. In this new study, we identified the different roles that *YKL-40* can play as a function of *MGMT* promoter methylation status and that these differential roles are involved in the *RAS–MEK–ERK* pathway in GSCs. The precise interaction between the *YKL-40* gene and *RAS–MEK–ERK* signaling requires further investigation.

In summary, our findings have emphasized the critical role of epigenetic status in cancer-related gene function. It also sheds light on the notion that gene expression quantification based on molecular tumor classifications should consider the epigenetic context in the future. The clinical implications of our work include: (1) the potentially beneficial role of combined gene targeting therapies based on the underlying epigenetic context; (2) the potential role that epigenetic context may have in cancer gene function in other human malignancies.

## Materials and methods

### Isolation and culture of WZs

Surgical specimens of GBMs were collected at Xijing Hospital by W.Z. (WZ series) with approval by the Institutional Review Board. Tissues were processed as previously described^30^. See Supplementary Material for details.

### Differentiation induction and immunocytochemistry

See Supplementary Material for details.

### Viral infections

See Supplementary Material for details.

### GBM pathology review, DNA extraction, and MGMT promoter methylation analysis

All tissue samples from primary tumors were confirmed by pathology review to represent GBM according to the World Health Organization classification of tumors of the central nervous system. MGMT promoter methylation was determined by MSP. See Supplementary Material for details.

### RNA isolation and quantitative real-time PCR

Total RNA was isolated from cultured cells using TRIzol (TaKaRa, Tokyo, JPN) and purified using miRNeasy columns (Qiagen). All RNA isolation and quantitative real-time PCR were performed according to our previous studies^1,2,20^. See Supplementary Material for details.

### ELISA for YKL-40 in culture medium

ELISA was performed according to our previous study^2^. See Supplementary Material for details for details.

### Western blotting analyses

Western blotting analyses were performed according to our previous study^1^. See Supplementary Material for details.

### Single-cell sphere-formation assay and BrdU

TMZ (Merck Sharp & Dohme Ltd, Whitehouse, NJ, USA) was dissolved in 5% dimethyl sulfoxide (DMSO) in phosphate-buffered saline (PBS). The doses of TMZ ranged from 0.01 to 1000 µM. Dose–response curves and effective dose (ED_50_) values were obtained and compared at day 5. See Supplementary Material for details.

### Inhibitor

The ERK pathway specific inhibitor PD98059 was purchased from Calbiochem (Millipore). See Supplementary Material for details.

### RNA-Seq and data analysis

RNA sample collection and preparation: total cellular RNA was extracted using TRIzol (TaKaRa, Tokyo, JPN). RNA degradation and contamination were monitored on 1% agarose gels. RNA concentration was measured using Qubit^®^ RNA Assay Kit in Qubit^®^ 2.0 Flurometer (Life Technologies, CA, USA). See Supplementary Material for details. Data generated for this study are available through the Gene Expression Omnibus (GEO: GSE153794).

### Identification of gene expression-based subtypes

We downloaded the Verhaak’s gene expression signatures of 840 genes (ClaNC840_centroids.xls)^6^ to determine the subtype of each sample according to four known molecular subtypes (neural, proneural, classical, mesenchymal). Training and prediction were performed using an R implementation of ClaNC software, a nearest centroid-based classifier. A training set consisting of 173 samples and 840 genes was used to predict subtypes in our samples as described by Verhaak^6^.

### Tumorigenicity studies

Female athymic nu/nu mice aged 6–8 weeks were obtained from the Fourth Military Medical University Experimental Center (Shaanxi, CHN) and were anesthetized with pentobarbital according to our previous study^1^. All animal procedures were performed with the approval of the Subcommittee on Research Animal Care (SRAC) at Xijing Hospital. To generate intracerebral xenografts, 1 × 10^5^ WZs in 2 µl of PBS were stereotactically implanted into the right cerebrum (2 mm lateral to the bregma at a depth of 3 mm) as previously described^20^. Mice were monitored and euthanized when they developed significant neurological symptoms. Formalin-fixed paraffin-embedded sections were stained with H&E. All animal procedures were performed with the approval of the SRAC at Xijing Hospital.

### Statistics

Comparisons of data obtained from real-time PCR, cell survival, single sphere-formation assay, and MGMT promoter methylation were performed using two-tailed Student’s *t* tests (unpaired). Survival analysis was performed with Kaplan–Meier curves, and their comparisons were examined with log-rank tests. *P* values < 0.05 were considered significant.

## Supplementary information

SUPPLEMENTAL MATERIAL

SUPPLEMENTAL Figure S1
