# Supplementary material for: The different role of YKL-40 in glioblastoma is a function of MGMT promoter methylation status
Source: Cell Death Dis. 2020 Aug 21;11(8):668. doi: 10.1038/s41419-020-02909-9 (PMC7441403; doi:10.1038/s41419-020-02909-9)
Supplement: Supplementary file 1 — SUPPLEMENTAL MATERIAL [file 41419_2020_2909_MOESM1_ESM.docx]

### Supplementary Materials and Methods

### Isolation and Culture of WZs

Surgical specimens of GBMs were collected at Xijing Hospital by Dr. Wei Zhang (WZ series) with approval by the Institutional Review Board. Tissues were processed as previously described [^1^](#_ENREF_1). Briefly, mechanically minced tissues were digested with 0.1% trypsin and 10 U/mL of DNaseI at 37°C for 45 min. After several washes, the tissues were triturated and passed through a 100-μm cell strainer. Cells were plated in EF medium consisting of Neurobasal medium (Invitrogen, Gaithersburg, MD, USA) supplemented with 3 mmol/L l-glutamine (Mediatech, Herndon, VA, USA), 1× B27 supplement (Thermo Fisher Scientific, Walthamm, MA, USA), 0.5× N2 supplement (Thermo Fisher Scientific), 2 μg/mL heparin (Sigma-Aldrich, St. Louis, MO, USA), 20 ng/mL recombinant human EGF (Peprotech, Rocky Hill, NJ, USA), 20 ng/mL recombinant human FGF2 (Peprotech), and 0.5× penicillin G/streptomycin sulfate/amphotericin B complex (Mediatech). Part of the digested tissue was also grown in DMEM supplemented with 10% FCS to generate standard primary adherent cultures. The cultures for GSCs were fed every third day with 1/3 volume of fresh medium. Passaging of the cultures was performed by dissociating the neurospheres using NeuroCult Chemical Dissociation kit (StemCell Technologies, Vancouver, BC, CAN).

### Differentiation Induction and Immunocytochemistry

To induce differentiation, the cells were grown in 10% FCS-containing medium devoid of EGF and FGF2 for 10 to 14 d. The cells were then fixed with 4% paraformaldehyde, permeabilized with 0.1% Triton X-100, washed, and blocked with 10% goat serum, prior to incubation with primary antibodies at 4°C overnight. Cell nuclei were visualized using 4’,6-diamidino-2-phenylindole (DAPI) (1:1000) (Ebioscience, San Diego, CA, USA) staining in adherent cells and Hoechst (1:1000) (Ebioscience) staining in neurospheres. The primary antibodies used were rabbit anti-GFAP (1:400) (Sigma-Aldrich), monoclonal mouse anti-β tubulin III (1:200) (Sigma-Aldrich), rabbit anti-nestin (1:200) (Santa Cruz Biotechnology, Dallas, TX, USA), and anti-CD133 (Millipore, Temecula, CA, USA, 1:500). FITC- or Cy3-conjugated secondary antibodies (1:1000) (Sigma-Aldrich) were applied at room temperature for 1 h to visualize immunoreactivity prior to observation. The stained cells were observed using fluorescent microscopy.

### Viral Infections

Y+ and Y- viral vectors were constructed and purchased from Cyagen Biosciences (Guangdong, CHN). Prior to transduction, we plated 2×10^5^ cells per well in a six-well plate with 2 ml EF medium. After six hours incubating at 37°C in a humidified 5% CO_2_ incubator, the appropriate virus was added to the cells. The medium containing the virus was gently mixed. Simultaneously, 5 μg/ml Polybrene (Cyagen Biosciences) was also added to the desired final concentration. After approximately 7 to 9 days in culture, individual spheres were observed and were visually scored for GFP/RFP-positivity. Spheres infected with empty vectors also expressed GFP/RFP as a control. The infected cells were selected using ampicillin.

### Glioblastoma pathology review, DNA extraction, and *MGMT* promoter methylation analysis

All tissue samples from primary tumors were confirmed by pathology review (Prof. Qing Li) to represent glioblastoma according to the World Health Organization (WHO) classification of tumors of the central nervous system. Tumor DNA was extracted from formalin-fixed and paraffin-embedded tissue samples using the Qiagen blood and tissue DNA extraction kit (Qiagen, Dusseldorf, GER). Each tumor sample used for DNA extraction was histologically confirmed to contain vital glioblastoma tissue with an estimated tumor cell content ≥80%. *MGMT* promoter methylation status was determined by methylation-specific PCR (MSP).

### RNA isolation and quantitative real-time PCR

Total RNA was isolated from cultured cells using TRIzol (TaKaRa, Tokyo, JPN) and purified using miRNeasy columns (Qiagen). Equal amounts of RNA were subjected to cDNA synthesis using the First Strand cDNA Synthesis Kit (Thermo Fisher Scientific) according to the manufacturer’s instructions. The cDNA expression was analyzed by quantitative reverse transcriptase polymerase chain reaction (qRT-PCR) using the SYBR Premix Ex Taq^TM^ II Assay kit (TaKaRa) under the following conditions: initial denaturation at 95°C for 3 min, followed by amplification for 40 cycles at 95°C for 30 s, 30 s annealing step at 60°C, 45 s elongation step at 72°C, and finalized with a 10 min elongation step at 72°C. We used the glyceraldehyde-3-phosphate dehydrogenase (GAPDH) primers (sense 5’-AAC GGA TTT GGT CGT ATT GGG C-3’ and antisense 5’-TAA GCA GTT GGT GGT GCA GG-3’) as a normalizing control. Other sequences of the primers applied in our research were designed based on the sequences of the genomic clones and are as follows: human YKL-40 sense: 5’-TGCAGCCAGAATGGGTGT-3’, antisense: 5’-CTGGTGTAGTAGCAGACCAGTTTGT-3’; human CD133 sense: 5’-AGTGGCATCGTGCAAACCTG-3’, antisense: 5’-CTCCGAATCCATTCGACGATA-3’; human Olig2 sense: 5’-TCGGAGCGAGCTCCTCAAAT-3’, antisense: 5’-GGGAAGATAGTCGTCGCAGCTT-3’; human Vimentin sense: 5’-TGACATTGAGATTGCCACCTACAG-3’, antisense: 5’-TCAACCGTCTTAATCAGAAGTGTCC-3’. The primer sequences used for the real-time quantitative PCR analyses are available upon request.

### ELISA for YKL-40 in Culture Medium

The amount of YKL-40 protein secreted by the cells was measured by collecting media samples and subjecting them to a two-site sandwich-type ELISA (R&D Systems, Minneapolis, MN, USA), according to the manufacturer’s instructions. Each time point was analyzed in triplicate. The protein concentrations were determined with an OD value, which was measured at 405 nm wavelength using a Bio-Rad Benchmark Microplate Reader.

### Western Blotting Analyses

Western blotting analyses were performed as previously described [^2^](#_ENREF_2) with primary antibodies against Vimentin (1:1000), Olig2 (1:500), YKL-40 (1:250) (R&D Systems), Nestin (1:500) (Santa Cruz Biotechnology), Ras (1:1000), Phospho-MEK1/2 (1:1000), MEK1/2 (1:1000), Phospho-ERK1/2 (1:2000), ERK1/2 (1:2000), MGMT (1:1000), β-actin (1:2000), β-tubulin (1:2000), Vinculin (1:1000) (Cell Signaling Technology, Beverly, MA, USA), γH2AX (1:500) (Millipore), phospho-ATM (1:1000), ATM (1:1000) (Rockland, Gilbertsville, PA, USA) and followed by incubation for 1 h at room temperature with a secondary antibody (HRP-conjugated anti-rat or anti-rabbit IgG 1:5000; Cell Signaling Technology). Immunoreactive bands were visualized using Enhanced Chemiluminescence (Amersham Pharmacia Biotech, Buckinghamshire, UK) and quantified with a LAS-1000 plus Gel Documentation System (Fujifilm, Tokyo, Japan).

### Single-Cell Sphere-Formation Assay and BrdU

For each condition, single cells were plated in 150 μl of serum-free medium in a 96-well plate. The sphere number/96-well plate was assessed after 14 days. The mean and SD of two biological replicates were calculated. In the serial sphere-forming assay, the same procedure was repeated for two additional passages. TMZ (Merck Sharp & Dohme Ltd., Whitehouse, NJ, USA) was dissolved in 5% dimethyl sulfoxide (DMSO) in PBS. The doses of TMZ ranged from 0.01 to 1000 µM. Dose-response curves and effective dose (ED_50_) values were obtained and compared at day 5. BrdU assays were performed according to the manufacturer’s recommendations (Roche, Basel, CH). The experiments were repeated at least three times for each of the conditions, and each experiment was performed in triplicate.

### Inhibitor

The ERK pathway specific inhibitor PD98059 was purchased from Calbiochem (Millipore). Prior to use, small molecules were dissolved in DMSO. For analysis of the following ERK pathway inhibitor treatment, all of the WZs were seeded at 2×10^5^ cells per well into a six-well plate, with indicated drugs (40 μM) added approximately 24 h prior to the final harvest.

### RNA-Seq

RNA sample collection and preparation: Total cellular RNA was extracted using TRIzol (TaKaRa, Tokyo, JPN). RNA degradation and contamination were monitored on 1% agarose gels. RNA concentration was measured using Qubit® RNA Assay Kit in Qubit® 2.0 Flurometer (Life Technologies, CA, USA). RNA integrity was assessed using the RNA Nano 6000 Assay Kit of the Bioanalyzer 2100 system (Agilent Technologies, CA, USA). Library preparation and sequencing: The poly-(A) enriched RNA sequencing libraries were prepared according to a previously published protocol, using 0.5 µg of total RNA per library in all instances. Dynabeads mRNA Purification Kit (Ambion, CA, USA) was used to isolate poly-(A) mRNA from total RNA. Random hexamer-primers were used to synthesize first-strand cDNA, while second-strand cDNA was synthesized using buffer, dNTPs, RNase, and DNA polymerase I. Then the double-stranded cDNA fragments were purified using 1.8×Agencourt AMPure XP Beads (Beckman Coulter, CA, USA). Suitable fragments were enriched via PCR amplification. All of the library preparations were multiplexed and sequenced on an Illumina Hiseq 2000 platform and 90 bp paired-end reads were generated.

### Data Analysis

Quality control: Raw data (raw reads) was firstly processed through Fastq-Mcf tool with defaults parameters (https://github.com/ExpressionAnalysis/ea-utils/blob/wiki/FastqMcf.md). Clean data (clean reads) were obtained by removing reads containing adapter, reads containing ploy-N and low quality reads from raw data. Reads mapping: Reference genome and gene model annotation files were downloaded from genome website directly. Index of the reference genome was built using Bowtie v2.1.0[^3^](#_ENREF_3) and paired-end clean reads were aligned to the reference genome using TopHat v2.0.12[^4^](#_ENREF_4)^,^[^5^](#_ENREF_5). TopHat was selected as the mapping tool for that TopHat can generate a database of splice junctions based on the gene model annotation file and thus a better mapping result than other non-splice mapping tools[^4^](#_ENREF_4). Quantification of gene expression level: Cufflinks version 2.2.1[^6^](#_ENREF_6)^,^[^7^](#_ENREF_7) was used to count the reads numbers mapped to each gene. And then FPKM of each gene was calculated based on the length of the gene and reads count mapped to this gene. FPKM, expected number of Fragments Per Kilobase of transcript sequence per Millions base pairs sequenced, considers the effect of sequencing depth and gene length for the reads count at the same time, and is currently the most commonly used method for estimating gene expression levels. Differential expression analysis: Differential expression analysis was performed using Cufflinks version 2.2.1. Genes with an adjusted *P*<0.05 found by Cufflinks were assigned as differentially expressed.

### Identification of Gene Expression-Based Subtypes

We downloaded the Verhaak gene expression signatures of 840 genes (ClaNC840_centroids.xls) [^8^](#_ENREF_8) to determine the subtype of each sample to four known molecular subtypes (neural, proneural, classical, mesenchymal). Training and prediction were performed using an R implementation of ClaNC software, a nearest centroid-based classifier. A training set consisting of 173 samples and 840 genes was used to predict subtypes in our samples as described by Verhaak RG [^8^](#_ENREF_8).

### Tumorigenicity Studies

Female athymic nu/nu mice aged 6 to 8 weeks were obtained from the Fourth Military Medical University Experimental Center (Shaanxi, CHN) and were anesthetized with pentobarbital. To generate intracerebral xenografts, 1 × 10^5^ WZs in 2 µl of phosphate-buffered saline (PBS) were stereotactically implanted into the right cerebrum (2 mm lateral to the bregma at a depth of 3 mm) as previously described [^9^](#_ENREF_9). Mice were monitored and euthanized when they developed significant neurological symptoms. Formalin-fixed paraffin-embedded sections were stained with H&E. All animal procedures were performed with the approval of the Subcommittee on Research Animal Care (SRAC) at Xijing Hospital.

### Statistics

Comparisons of data obtained from real-time PCR, cell survival and single sphere formation assays were performed using two-tailed Student's t-tests (unpaired). Survival analysis was performed with Kaplan-Meier curves, and their comparisons were examined with log-rank tests. P-values <0.05 were considered significant.

**References**

1 Wakimoto, H. et al. Human glioblastoma-derived cancer stem cells: establishment of invasive glioma models and treatment with oncolytic herpes simplex virus vectors. Cancer research 69, 3472-3481, doi:10.1158/0008-5472.CAN-08-3886 (2009).

2 Zhang, W. et al. Association between YKL-40 and adult primary astrocytoma. Cancer 116, 2688-2697 (2010).

3 Langmead, B., Trapnell, C., Pop, M. & Salzberg, S. L. Ultrafast and memory-efficient alignment of short DNA sequences to the human genome. Genome Biol 10, R25, doi:10.1186/gb-2009-10-3-r25 (2009).

4 Trapnell, C., Pachter, L. & Salzberg, S. L. TopHat: discovering splice junctions with RNA-Seq. Bioinformatics 25, 1105-1111, doi:10.1093/bioinformatics/btp120 (2009).

5 Kim, D. et al. TopHat2: accurate alignment of transcriptomes in the presence of insertions, deletions and gene fusions. Genome Biol 14, R36, doi:10.1186/gb-2013-14-4-r36 (2013).

6 Trapnell, C. et al. Transcript assembly and quantification by RNA-Seq reveals unannotated transcripts and isoform switching during cell differentiation. Nat Biotechnol 28, 511-515, doi:10.1038/nbt.1621 (2010).

7 Trapnell, C. et al. Differential analysis of gene regulation at transcript resolution with RNA-seq. Nat Biotechnol 31, 46-53, doi:10.1038/nbt.2450 (2013).

8 Verhaak, R. G. et al. Integrated genomic analysis identifies clinically relevant subtypes of glioblastoma characterized by abnormalities in PDGFRA, IDH1, EGFR, and NF1. Cancer Cell 17, 98-110, doi:10.1016/j.ccr.2009.12.020 (2010).

9 Zhang, W. et al. Bevacizumab with angiostatin-armed oHSV increases antiangiogenesis and decreases bevacizumab-induced invasion in U87 glioma. Mol Ther 20, 37-45, doi:10.1038/mt.2011.187 (2012).

**Figure S1. Secretion levels of *YKL-40* by GSCs**

ELISA assays were performed to quantify the protein secretion levels of *YKL-40* in GSCs.
