# Supplementary figures and images for: The different role of YKL-40 in glioblastoma is a function of MGMT promoter methylation status
Source: Cell Death Dis. 2020 Aug 21;11(8):668. doi: 10.1038/s41419-020-02909-9 (PMC7441403; doi:10.1038/s41419-020-02909-9)

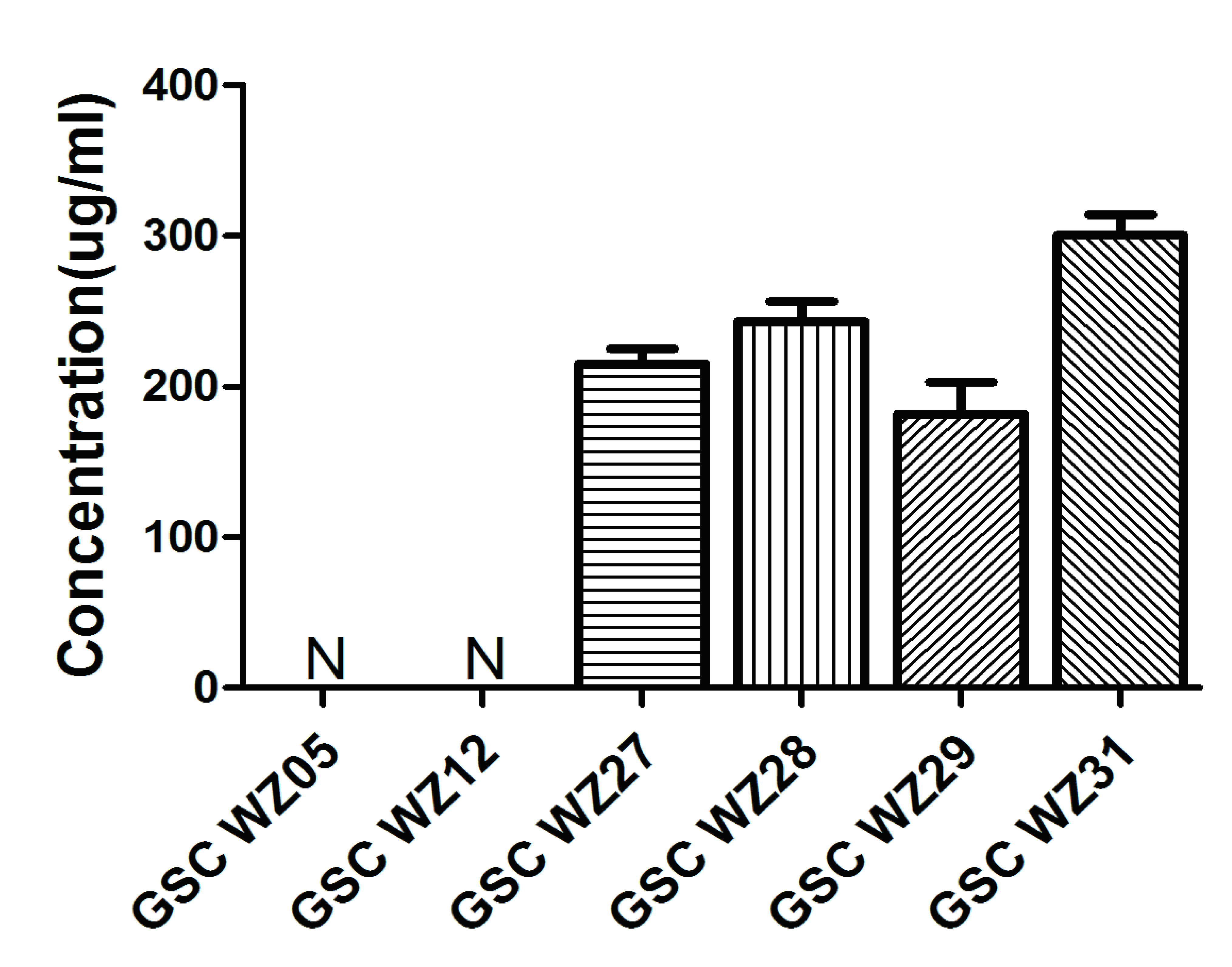

Supplement: Supplementary file 2 — SUPPLEMENTAL Figure S1 [file 41419_2020_2909_MOESM2_ESM.tif]
